# Geographical evolutionary pathway of global tuberculosis incidence trends

**DOI:** 10.1186/s12889-023-15553-7

**Published:** 2023-04-24

**Authors:** Yanhui Lei, Jinfeng Wang, Yang Wang, Chengdong Xu

**Affiliations:** 1 State Key Laboratory of Resources and Environmental Information System, Institute of Geographic Sciences and Natural Resources Research, Chinese Academy of Sciences, Beijing, 100101 China; 2 University of Chinese Academy of Sciences, Beijing, 100049 China

**Keywords:** Tuberculosis, Stratified heterogeneity, Geographical evolutionary pathway, Geotree

## Abstract

**Backgrounds:**

Tuberculosis (TB) remains a serious public health and human development problem, especially in developing countries. Despite the effectiveness of directly observed therapy, short course programs in reducing transmission and progression of TB, poverty reduction and socioeconomic development remain crucial factors in decreasing TB incidence. However, the geographical pathway on the planet is not yet clear.

**Objectives:**

This study was to reconstruct the geographical evolutionary process of TB in 173 countries and territories from 2010 to 2019 to analyze the socioeconomic determinants that impact the global TB epidemic. In addition, the TB incidence in 2030 was predicted.

**Methods:**

This study analyses TB incidence data from 173 countries and territories between 2010 and 2019. The Geotree model would be used to reconstruct the geographical evolutionary process of TB, which provides a simplified schema for geo-visualizing the trajectories of TB incidence and their socioeconomic drivers. Additionally, to estimate the future TB incidence in 2030, a multilevel model was utilized in conjunction with the hierarchical nature of the Geotree based on a stratified heterogeneity analysis.

**Results:**

Global TB incidence was found to be associated with the country type and development stages. Between 2010 and 2019, the average TB incidence rate in 173 countries and territories was -27.48%, with marked spatially stratified heterogeneity by country type and development stage. Low-income and lower-middle-income countries were most vulnerable to TB. Upper-middle-income countries experienced a faster decline in TB incidence than high-income countries, and TB incidence generally decreased as the development stage increased, except for the lower-middle development stage in 2019.The highest average rate of decline in TB incidence was observed in the upper-middle development stage of high-income countries, with a reduction of 45.24%. Meanwhile, 37 high-income countries in the high development stage demonstrated an average rate of change of -13.93%. Socioeconomic determinants, including gross domestic product per capita, urbanization rate, and sociodemographic index, were found to inhibit TB incidence. Based on current trends, the predicted average global TB incidence in 2030 is 91.581 per 100,000 population.

**Conclusions:**

The trajectories of the global TB incidence have been reconstructed to formulate targeted public health responses. To eliminate TB, countries at similar development stage can draw on the experiences of countries at higher development stages that are tailored to their unique characteristics. By learning from successful TB control strategies, countries can take strategic steps toward eradicating TB and improving public health outcomes.

**Supplementary Information:**

The online version contains supplementary material available at 10.1186/s12889-023-15553-7.

## Background

As a major cause of human illness and death, tuberculosis (TB) remains one of the world’s principal infectious diseases, particularly affecting poor and susceptible populations [1]. The End TB Strategy of the World Health Organization (WHO) and the sustainable development goals (SDGs), which aim to reduce the number of TB deaths from TB by 90% and the incidence rate of TB by 80% from 2015 to 2030, have promising targets for ending the TB epidemic [2–4]. In 2019, an estimated 10 million individuals worldwide were infected with TB, and 1.2 million deaths among HIV-negative individuals were from TB and 208,000 deaths among HIV-positive individuals were attributed to TB.

TB is prevalent in all regions of the world and has become a major problem in low-income and middle-income countries’ health burden [5–7]. An understanding of the evolutionary pathway of the incidence rate of TB and the burden of TB is crucial for evaluating the progress towards ending the epidemic and for informing policy and programs at preventing TB.

The SDG milestones for 2020 were not fulfilled [8]. Progress towards universal health coverage and progress in the actions addressing the social and economic problems of health is needed to fulfill these SDG targets. The development and transmission of TB mainly depend on socioeconomic determinants [9]. The SDG target framework rejects a one-size-fits-all solution to fulfill the targets and argues for greater attention to the geospatial process that underscores regional differences. Thus, comparing the observed TB burden with the expected TB burden based on a country’s sociodemographic profile could be helpful in guiding investment in research and interventions [10, 11]. Strategies to effectively address the TB burden are needed.

Many previous studies have used multivariate regression models and the Bayesian method to determine the global relationships between the prevalence of TB and selected factors [12–15]. However, it is difficult to incorporate a spatial stratified heterogeneity analysis of the effects using these traditional models. Identifying stratified heterogeneity in the spatial distribution of TB cases and characterizing its drivers can help inform targeted public health responses, making this an attractive approach. Spatial dependence and heterogeneity have been observed in the study of analyzing TB incidence, but they are also less efficient in representing and visualizing the geospatial temporality in a simple schema [16]. Thus, exploring the geographical evolutionary pathway of TB based on its stratified heterogeneity is urgently needed to monitor the effectiveness of TB control strategies and programs.

Geotree, a new multidimensional visualization and analysis model with stratified heterogeneity, was first introduced by Wang et al. [17] to analyze the evolutionary pathways of growing cities and predict urban growth in a ‘‘tree’’ structure, with the city types grouped as branches and the leaf position along the branches reflecting the development stage. The Geotree model is not limited by dimensions. By combining the law of development, the mechanisms and evolution that may exist in multidimensional data can be expressed in a simple and clear visual form.

The Geotree is also a new type of coordinate system that presents attribute and geographical spaces by combining it with a map. Therefore, we attempted to conceptualize and examine the geospatial temporal processes affecting the TB incidence trend by combining the Geotree with the designation of the countries into country types and development stages.

We assumed that there is an inter-regional correlation between the TB incidence trend and its socioeconomic drivers and that the tree structure of Geotree can efficiently organize the spatial analysis of the TB incidence trend such that multidimensionality can be not only visualized but also quantified and conceptualized through cross-sectional data [18].

This study was conducted to explore the evolutionary pathway of TB, which can be explained by different socioeconomic settings in Geotree based on a stratified heterogeneity analysis to geo-visualize the trajectories and relevant socioeconomic determinants, for the purpose of providing essential information for the control and prevention of TB. Geotree.

### Socioeconomic determinants of TB

Several studies have examined the determinants of TB [19–21]. Fully understanding the compositional and contextual drivers of TB is helpful for formulating targeted measures and strategies for TB control and prevention. The occurrence of TB is a consequence of multiple socioeconomic factors, including individual- and ecological-level factors.

Poverty [19, 22–24] and socioeconomic development [24, 25] are key determinants that are intertwined with TB infection and disease. At the individual level, individual differences in education [26], drinking [27], smoking [28], and related diseases [29, 30] are common drivers that influence the burden of TB. At the ecological level, socioeconomic determinants [31, 32] play a key role in determining the burden of TB. These socioeconomic determinants include the national economic level [33], unemployment rate [27], urbanization rate [34], poverty rate [22, 23] and social instability [35] of a country. In this study, socioeconomic determinants that can reduce the burden of TB were used to analyze the development of TB (Supplementary file 1. Fig. S1).

The evolutionary pathway of TB incidence based on stratified heterogeneity is unclear yet. To address this knowledge gap, this study aims to reconstruct the evolutionary pathway of TB incidence and investigate the relationship between TB incidence and changes in socioeconomic development on a global scale. This study also examined how TB incidence evolves in response to changes in country types and development stages and predicts TB incidence rate in 2030 by using the stratified heterogeneity of the evolution tree.

## Methods

This study aims to utilize the Geotree model to reconstruct the geographical evolutionary process of TB across 173 countries and territories. The study analyzes TB incidence rates between 2010 and 2019, categorized by country type and development stage. Additionally, the study estimates the TB incidence rate in 2030, utilizing a multilevel model combined with the hierarchical nature of the Geotree. By visualizing trajectories and socioeconomic drivers that affect global TB incidence, the results of this study can inform targeted public health responses to eliminate TB.

### TB incidence data

In this study, we undertook an analysis of global TB incidence data in 173 countries and territories in 2016 and 2019. The data on the TB incidence rate were sourced from the WHO database [36], where the TB incidence has already been collected.

### Socioeconomic data

The WHO Commission on Social Determinants [37] defines structural health determinants as conditions that generate or reinforce social stratification in society [25]. Similarly, social stratification results in an unequal distribution of social determinants. Thus, these factors account for the unequal distributions of the key social determinants of TB. In this study, socioeconomic determinants were used to analyze the development of TB ((Supplementary file 1. Fig. S1). These socioeconomic determinants include the national economic level, health access, education, income level, and urban populations (Supplementary file 1. Fig. S1).

#### GDP per capita

Considering economic development, GDP per capita was a proxy factor for a country’s economic development and was used to define the type of country, which was sourced from the World Bank database [38].

#### Socio-demographic index

The socio-demographic index (SDI) [39], which is based on the geometric mean of three indicators (income per capita, average years of schooling, and total fertility rate), was developed for the Global Burden of Disease Study (GBD) 2015 [39], and was updated for GBD 2016 [40–42]. The SDI scores were scaled from 0 to 1, and each location had an SDI score for each year. The SDI was used to help generate a “tree” to explore the geographic evolutionary pathways of TB.

In terms of social development, the SDI [43] was used to divide the 173 countries and regions into four SDI levels—low, low-middle, middle, high-middle, and high.

#### Urbanization rate

The prevalence of TB is higher in urban areas than in rural areas [34]. The urbanization level is a criterion of urbanization development and was used to define the development stage of a country [44, 45]. The urbanization rate for each country was sourced from the World Bank database [46].

#### Future data

In this study, the data used in the MLM combining with Geotree model to predict the prevalence of TB in 2030 were obtained from the Shared Socioeconomic Pathways (SSP) database [47], including the data on the global urbanization rate and global GDP in the middle of the road (SSP2), which were selected as the explanatory variables in the prediction model.

The SDI data were linearly extrapolated from the 1990–2019 data to obtain SDI data for countries for 2030, which were used as explanatory variables in the MLM.

### Geotree

Geotree [17] model has progressed rapidly in the past few decades and can better analyze the underlying evolutionary pathway of TB based on the stratified heterogeneity, which draws on the biological theory [48] of evolution. The Geotree is categorized with the TB incidence using different leave colors, with a “tree” structure with the country types grouped as the branches, the leaf position along branches in an orderly orientation reflecting the country development stages, and the leaves representing the countries (Supplementary file 1. Fig. S2). In the Geotree, the first branches and the second trunks are annotated with colors corresponding to the locations of the country types and country development stages, and each leaf is labeled with the country code (Supplementary file 1. Fig. S2).

#### Country types (First branches)

In this study, 173 countries and territories were divided into four different country types according to the World Bank’s 2018 income standards, which included low-income countries (I), lower middle-income countries (II), upper middle-income countries (III), and high-income countries (IV). The countries within each country types are listed in Supplementary file 1. Table S1 and Supplementary file 1. Fig. S3.

#### Country development stages (Second trunks)

The 173 countries and territories were divided into five development stage: low (1), lower-middle (2), middle (3), upper-middle (4), and high (5), using a measurement for the indicators of the SDI and urbanization rate (Supplementary file 1. Table S2). Then, a weighting method was used to integrate the two divisions, and the weights were measured by the explanatory power of TB incidence using GeoDetector [49, 50], with the values for the SDI and urbanization rates being 0.58 and 0.42 in 2010, respectively, and, 0.63 and 0.37 in 2019, respectively. The countries for each of the development stages, 1 to 5, in 2010 and 2019 are listed in Supplementary file 1. Table S3 and Supplementary file 1. Table S4.

### Multilevel model

The multilevel model (MLM) [51, 52] was used to combine the stratified structures and of the TB incidence Geotree to predict TB incidence rate in 2030. The MLM extended the general regression by analyzing stratified [52, 53] and cross-classified data [52], and it examined the effects of group- and individual-level covariates on individual-level outcomes. The cross-classified MLM was used in this study, and the countries were cross-classified by the country type and development stage. The formula for cross-classification model is as follows:

1 $${y}_{i(t,s)}={\beta }_{0}+{\beta }_{1}{x}_{i(t,s)}+{u}_{t}+{u}_{s}+{e}_{i\left(t,s\right)} {u}_{t}\sim N(0,{\sigma }_{u(t)}^{2}),{u}_{s}\sim N(0,{\sigma }_{u(s)}^{2}){e}_{i(t,s)}\sim N(0,{\sigma }_{e}^{2})$$

where ${y}_{i(t,s)}$ is the parameter of the TB incidence rate of country $i$, contained in the leaf of Geotree defined by the type of country type $t$ and development stage $s$. In addition, ${\beta }_{0}$ is the mean TB incidence rate across all group-level units (i.e., country type and development stage); ${u}_{t}$ and ${u}_{s}$ are the effects of country type $t$ and development stage $s$, respectively; and ${e}_{i(t,s)}$ is the country-level residual error term.

### Geodetector method

The explanatory power of the factors influencing the TB incidence, and the stratified heterogeneity of TB incidence was detected using Geodetector’s q-statistic value [49, 50, 54]. The q-value is [0,1]. The higher the q-value, the higher the explanatory power. The formula used is as follows:

2 $$q = 1- \frac{\sum_{h=1}^{L}{N}_{h}{\sigma }_{h}^{2}}{N{\sigma }^{2}} =\frac{\sum_{h=1}^{L}\sum_{i=1}^{{N}_{h}}{({Y}_{hi}-\overline{{Y }_{h}})}^{2}}{\sum_{i=1}^{N}{({Y}_{i}-\overline{Y })}^{2}}$$

TB incidence is grouped into *N* and stratified into *h* = *1,2,…, L* stratum; *Y*_*i*_ and *Y*_*hi*_ denote the TB incidence of country *i* in the population and in stratum *h*; $\overline{Y }$ and $\overline{{Y }_{h}}$ indicate the stratum and population means of the TB incidence rate; and ${\sigma }_{h}^{2}$ and ${\sigma }^{2}$ indicate the stratum variance and population variance, respectively.

## Results

### Stratified heterogeneity of the TB incidence

**Fig. 1:**
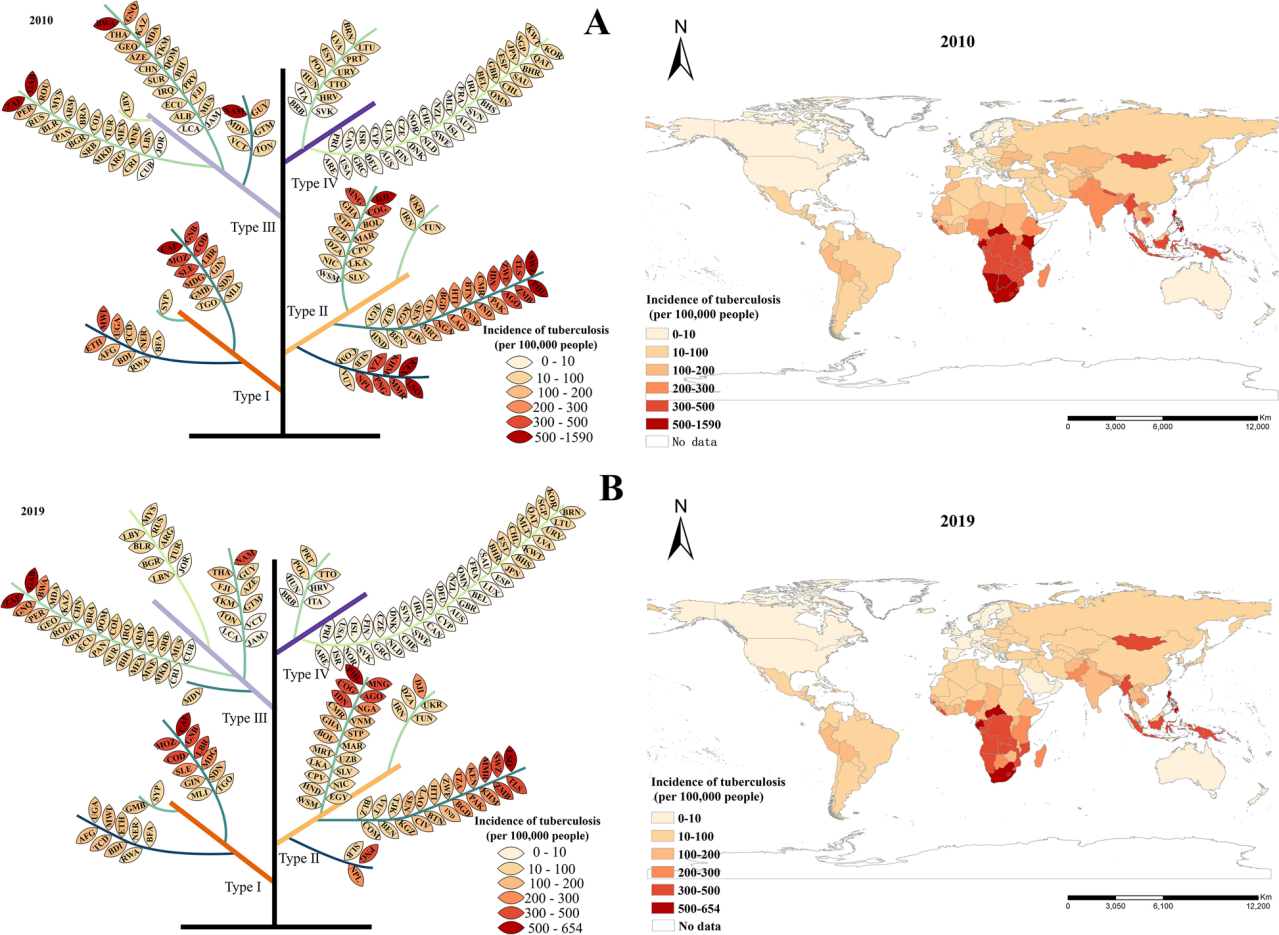
The TB incidence rate (per 100,000 population) Geotrees corresponding to the geospatial distributions in 2010 (**A**) and in 2019 (**B**) in 173 countries and territories

The TB incidence varied among the spatial distribution patterns (Fig. 1). For example, the low-income and lower-middle-income countries were more vulnerable to TB. In highly developed countries, the development of TB was relatively low. The q value represents the stratified heterogeneity of the TB incidence among the stratified countries under the different country division strategies. A higher q value indicates a greater degree of stratified heterogeneity of the TB incidence between the stratified countries.

In 2010, the country type had a q value of 0.198, and the country development stage had a q value of 0.214. The stratified heterogeneity of the TB incidence in different countries increased over time. In 2019, the country type had a q value of 0.277, and the country development stage had a q value of 0.29. All the q values were statistically significant. This implies that considering the country type and development stage simultaneously allows the division of a heterogeneous TB incidence into a more homogeneous stratification. Hence, the country type and development stage were selected as the best conditions for division to generate the TB incidence Geotree.

### TB incidence Geotree

The 173 countries and territories were divided into four types and five development stages, which were indicated by four branches and five trunks, respectively, in a “tree” structure. As seen in the TB incidence Geotree (Fig. 1), the geographic evolutionary pathway of the TB incidence from 2010 to 2019 trend was geo-visualized. In general, the TB incidence tree withered as the average TB incidence rate from 145 to 106 (per 100,000 population) over the study period, that is, tree branches grew in height as several countries progressed towards a higher development stage.

Notably, the leaves became denser at the top of each trunk as the countries entered a higher development stage in 2019. The growth of a new leaf can be predicted based on the current state of the tree structure. The evolutionary pathway of a tree can be described by the visualization of the tree branches, tree trunks, and tree leaves.

**Fig. 2:**
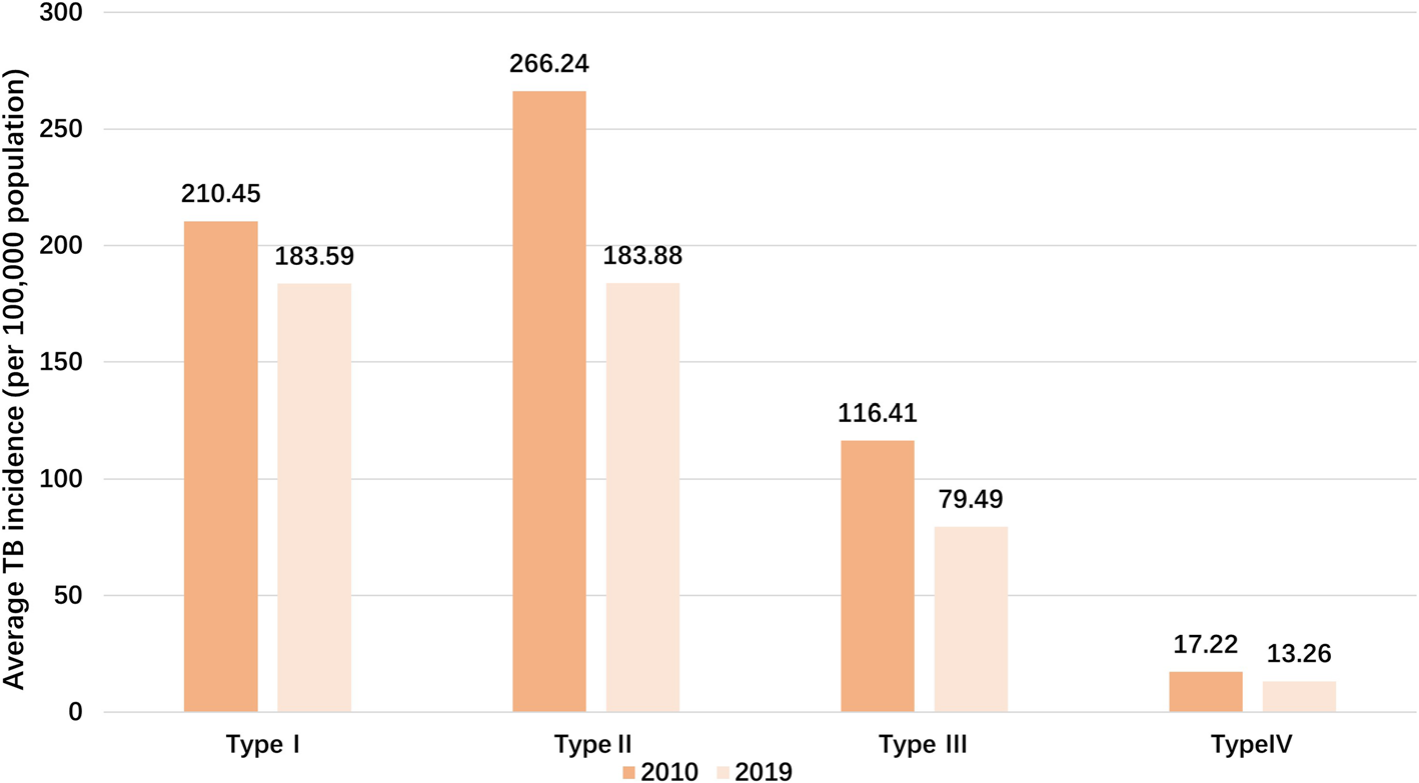
Average incidence of TB (per 100,000 population) in 2010 and 2019 for the four country types

First, the average TB incidence rate in 2019 was lower than that in 2010. It also can be deduced from the tree and the map (Fig. 1A and B) that, the incidence rate in 2010 in some countries exceeded 1000 per 100,000 population, such as in Lesotho, South Africa, and Eswatini. However, the highest TB incidence rate in 2019 in the study area was less than 700 per 100,000 population. Second, the branches of the type III countries (upper-middle- income countries) and type IV countries (high-income countries), which on the taller capture healthier, indicating a lower TB incidence rate (Fig. 1A and B; Fig. 2), both in 2010 and 2019.

The GDP per capita was highest for the type IV countries (high-income countries), which indicates that a lower TB incidence is accompanied by increasing affluence. In contrast, the average TB incidence rates in the low-income and lower-middle-income countries were higher and were accompanied by poverty. However, the average TB incidence rate in the type I countries (low-income countries) was slightly inferior to that in the type II countries (lower-middle-income countries) (Fig. 2).

**Fig. 3:**
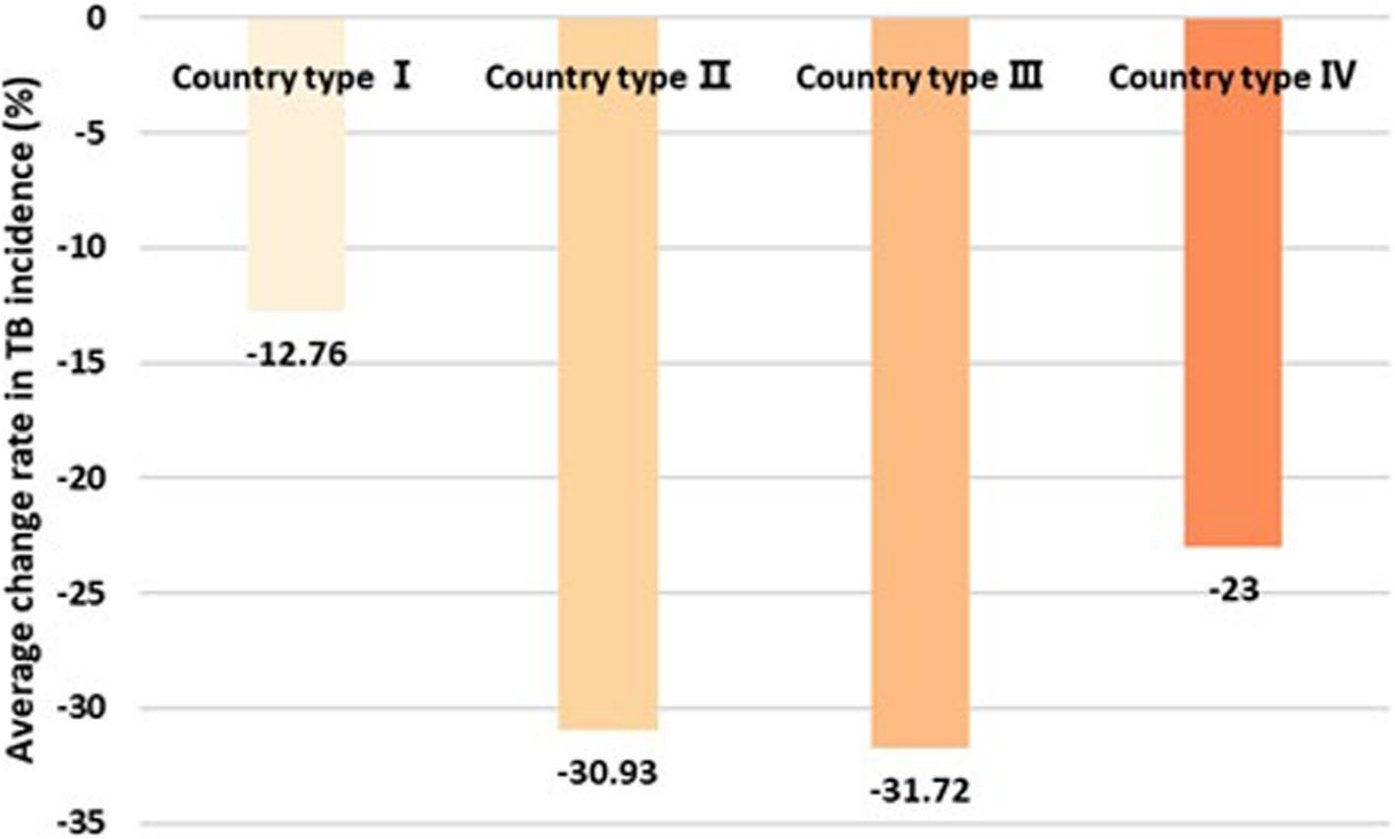
Average rate of change in the TB incidence (%) in 173 countries and territories from 2010 to 2019 for the four country types. Note: The countries in each country types are listed in Supplementary file 1. Table S1

On average, average rate of change in the TB incidence in type II countries (lower-middle-income countries) and type III countries (upper-middle-income countries) were the highest (average change rates: -30.93% and -31.72%, respectively; Fig. 3) from 2010 to 2019. When the countries within each country type were compared, the type I countries (low-income countries) showed a minimal average change in the TB incidence of -12.76 (Fig. 3).

**Fig. 4:**
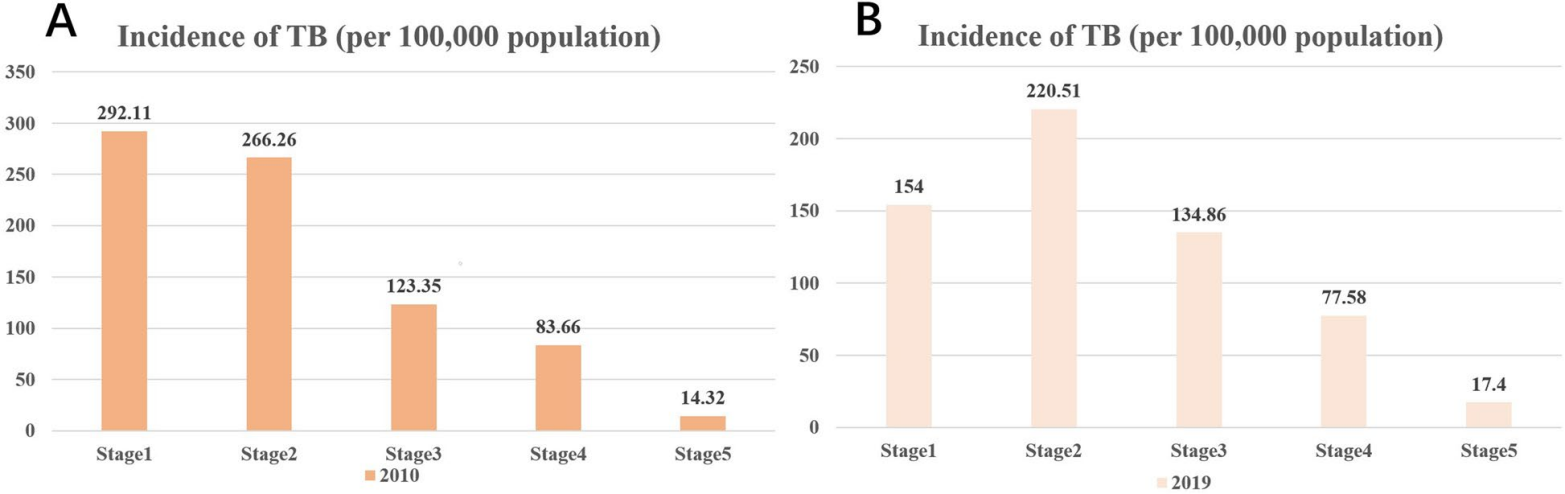
Average incidence of TB (per 100,000 population) in 2010 (**A**) and 2019 (**B**)for the five country development stages

**Fig. 5:**
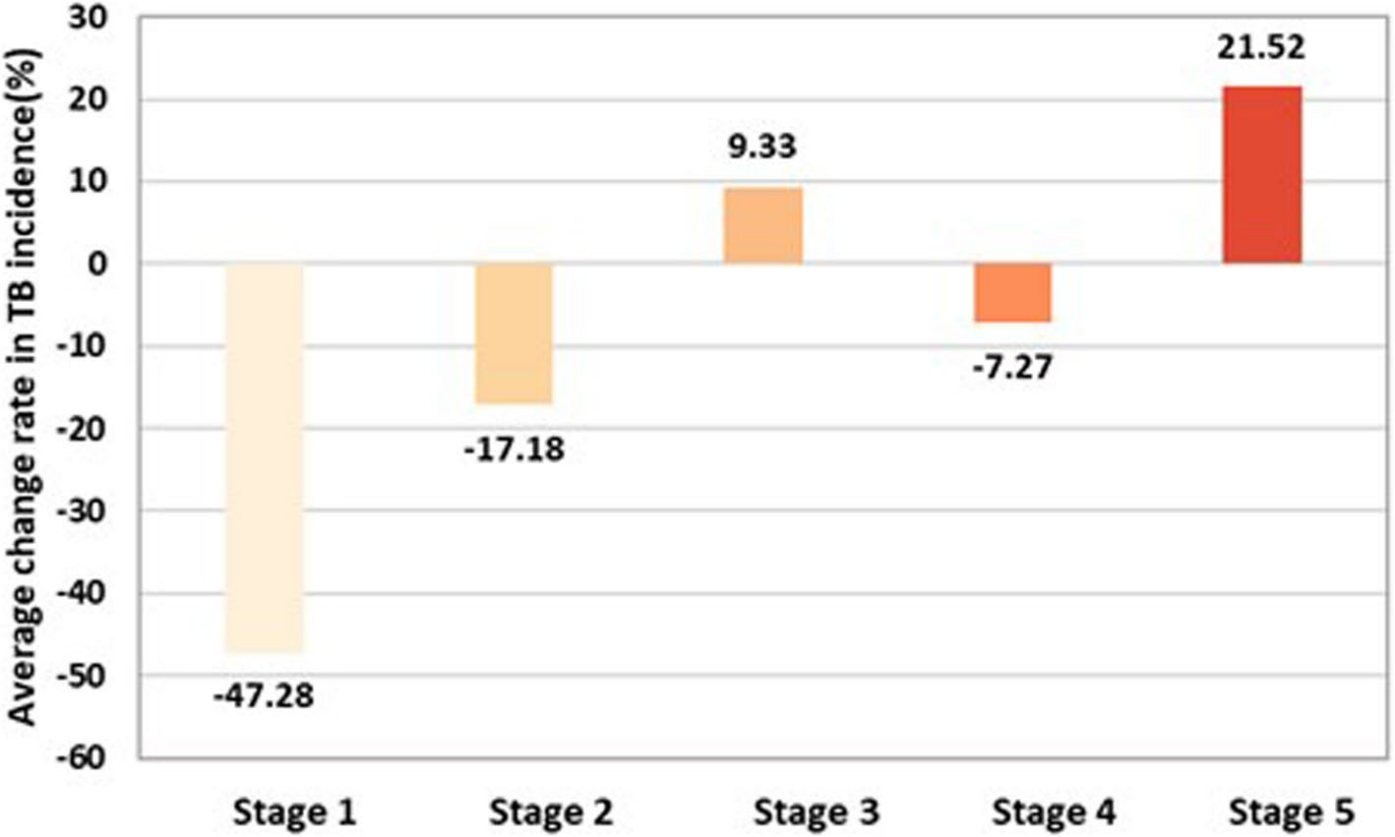
Average rate of change in the TB incidence (%) from 2010 to 2019 in 173 countries and territories based on the country development stage. Note: The countries in each development stages are listed in Supplementary file 1. Table S3 and Supplementary file 1. Table S4

Third, the average incidence of TB according to the country development stages exhibited different trends in 2010 and 2019. As shown in Fig. 4, the average TB incidence decreased as the country development stage increased, except for stage 2 in 2019. On average, the countries in development stage 1 exhibited the fastest decline: -47.28%, and the average rate of change in the TB incidence increased for development stage 3 and development stage 5 (average change rates: 9.33% and 21.525%, respectively; Fig. 5). When countries within development stages were compared, the countries in the development stage 4 exhibited a minimal average change in the TB incidence (-7.27%; Fig. 5).

Finally, one such characteristic of Geotree model is combing the map with the “tree” structure. Some phenomena appear random in the geographical distribution but appear as clear patterns in the attribute space. Combining the geographical distribution of the TB incidence with the evolutionary Geotree sheds light on the phenomenon of “hot spots” and spatial stratified heterogeneity around countries, as shown in Fig. 1.

At the bottom of the TB Geotree, a high TB incidence rate is shown in South Africa, the Philippines, Lesotho, Gabon, and Central African Republic, which were highest in 2010 and 2019, with TB incidence rates exceeding 500 per 100,000 population. In these countries and territories, the average TB incidence decreased from 808 to 577 (per 100,000 population) from 2010 to 2019.

### Driving socioeconomic factors of TB incidence

The socioeconomic level, described by country type and stage, which is calculated by the GDP per capita, urbanization rate, and SDI, is negatively correlated with TB incidence (Table 1). In general, the incidence of TB worldwide has decreased with economic growth [55, 56].

**Table 1:** Correlation analysis of the socioeconomic drivers of TB incidence

TB incidence	GDP per capita	Urbanization rate	SDI
2010	-0.321**	-0.38**	-0.411**
2019	-0.392**	-0.375**	-0.508**

^***^P < 0.01; **P < 0.05

The driving socioeconomic factors of TB incidence were detected using GeoDetector. The SDI, GDP per capita, and urbanization rate had a significant impact on the spatial pattern of the TB incidence, and their driving forces were 0.216, 0.198, and 0.156 in 2010, and 0.292,0.277, and 0.175 in 2019, respectively. This means that 21.6%, 19.8%, and 15.6% of the spatial stratification heterogeneity of the TB incidence in 2010 can be explained by the above mentioned three factors, respectively. Likewise, the above mentioned four factors can explain 29.2%, 27.7%, and 17.5% of the spatial stratification heterogeneity of TB incidence.

### Evolutionary pathway of the TB incidence trend

Countries of the same type and the epitaxial growth of a region, whose economic development is at a lower stage can follow the evolutionary path of the region that has transitioned into a higher development stage. The Markov chain analysis of the evolution of the country development stage, which compensates for time, is used to quantify the rate of change in the regional TB incidence.

**Fig. 6:**
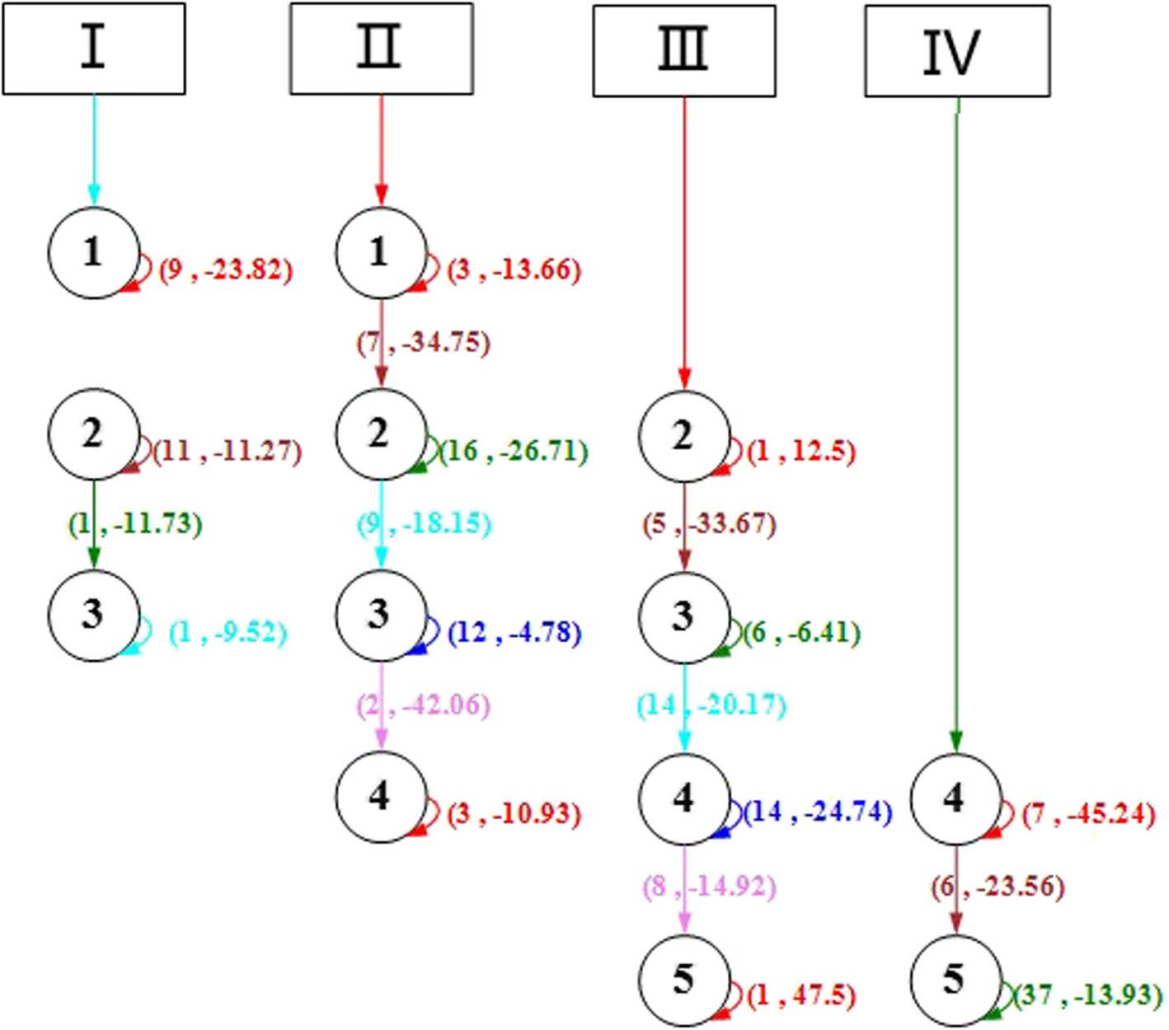
Evolutionary pathway of the country development stage by Markov chain analysis. Note: Each arrow represents a transition type, for which the tail and head of the arrow indicate the initial and final development stage levels, respectively. For each country type, the areas with the same initial and final country development stage are classified into one category for the statistical analysis, statistical data in the form of probability. The number of countries for which the development stage changed and the average rate of change in the TB incidence are shown in brackets near the arrow

The transition to the development stage is shown in Fig. 6. As shown in Fig. 6, the average rate of change in the TB incidence during the transition of city stages is rapid, with the highest average rate of change in the TB incidence being -45.24% in stage 4 of type IV countries and -42.06% from stage 3 to stage 4 in type II countries from 2010 to 2019, respectively. The analysis of 37 countries exhibited an average rate of change in the TB incidence of -13.93% in stage 5 of type IV countries. Socioeconomic progress dampened the increase in the TB incidence. However, only Libya had a staggering 55% increase in the TB incidence in 2010, which belongs to country development stage 5 of type III. It is also worth mentioning that the transition from development stage 1 in 2010 to development stage 2 in 2019 did not occur in any of the type I countries. The Markov chain represents the change in the city development stage corresponding to that of the evolutionary Geotree of the TB incidence in Fig. 1.

### Future prospects of the TB incidence

Embedding the MLM model into Geotree model can help complete the evolutionary path to predict the TB incidence in the future. The Geotree of TB incidence presents a hierarchical structure which can be introduced into the MLM model to further explore future trends of the TB incidence. The TB incidence in 2030 was predicted on the basis of hierarchical results (country types and country development stages) of the Geotree constructed using the 2019 TB incidence.

Global linear regression (GLR) without strata was used to validate the performance of the MLM. In the model fitting, the data were randomly divided into training data (70%) and test data (30%). Table 2 compares the performances of the two models and indicates that the MLM performs better than the GLR.

**Table 2:** Overall accuracy of the MLM and the GLR model results

	RMSE	MAE	R^2^
MLM	115	73.2	0.311
GLR	121	73.3	0.257

**Fig. 7:**
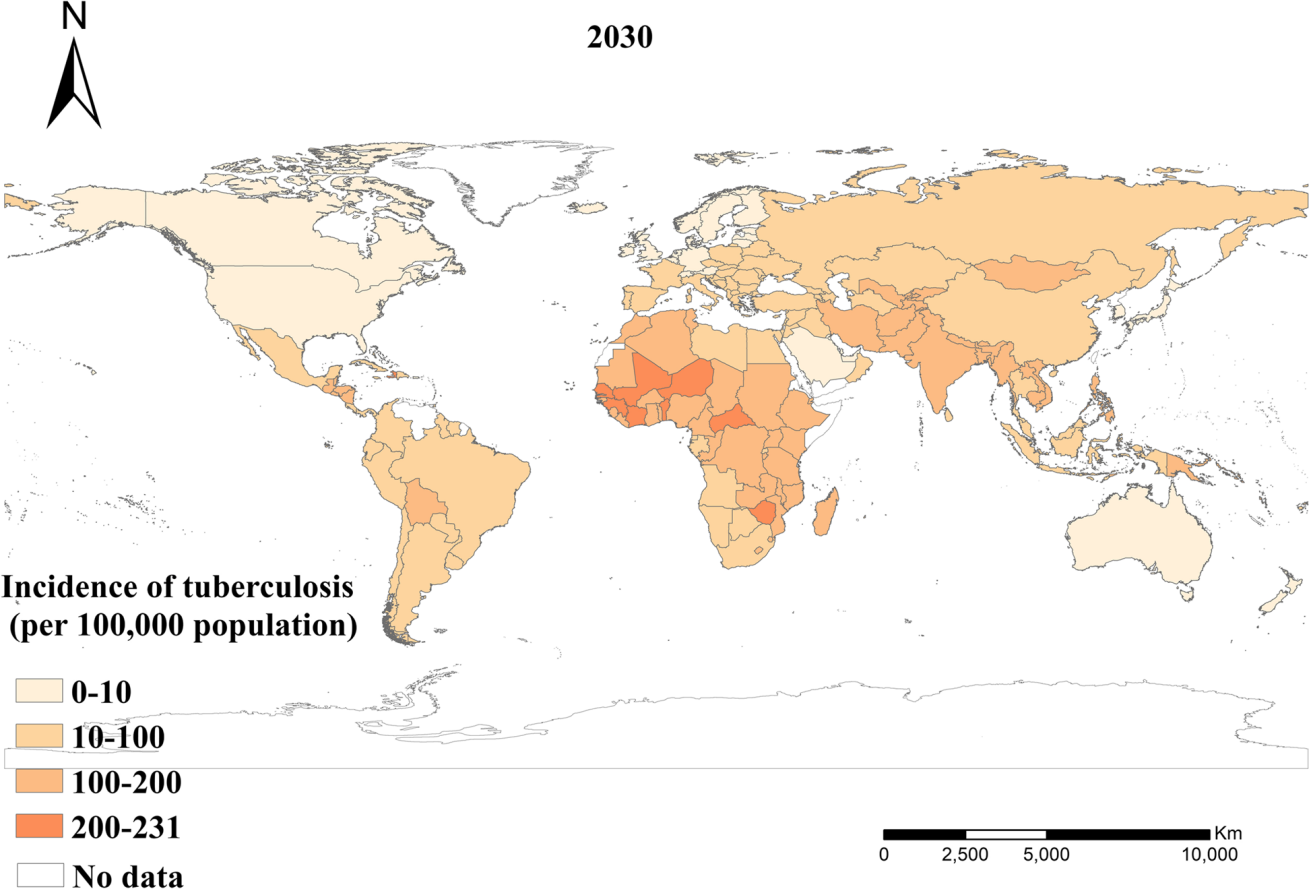
Prediction of the spatial TB incidence in 173 countries and territories in 2030

Using the trained MLM, we predicted the urban expansion rates of the 173 countries from 2019 to 2030. The results are presented in Fig. 7 and Supplementary file 2. Table S1. It is predicted that, by 2030, the average global TB incidence rate will be 91.581 (per 100,000 population). Based this forecast, there would be a significant reduction in the overall incidence rate in 2030. The incidence rate generally decreases at each stage of development, with lower development stage countries experiencing the highest incidence rates and high development stage countries having the lowest incidence rates. Specifically, the incidence rates are highest in low-middle development stage countries. By this estimation, 26 of 173 countries would eradicate TB. Of these, 20 out of 26 countries are in the development stage 5 of the country type IV. However, this result still does not fulfil the “End” “TB” target.

## Discussion

This study explored the evolutionary pathway of the global TB incidence in 173 countries and territories in 2010 and 2019 and adopted the Geotree model in combination with statistical methods to conceptualize and examine the geospatial temporal processes of global TB disease. The past and future mechanisms and evolution path of the TB incidence index can be visualized in a tree, which holds great significance to informing targeted public health responses.

The global TB incidence was associated with the country type and development stage based on a stratified heterogeneity analysis (Fig. 1); therefore, the geographical evolutionary process of TB was reconstructed by the Geotree model, with first branches and second trunks, which provides a simplified schema for geo-visualizing the trajectories and relevant socioeconomic drivers that affected the global TB incidence. The indicators for the TB incidence Geotree classification used in this study included the urbanization rate, GDP per capita, and SDI, which are all indicators for development. These socioeconomic determinants measure a country’s level of development from three aspects: (1) urbanization, (2) economics, and (3) sociodemographics (income level, medical, and education), which are also convenient for exploring the future prospects of the TB incidence.

Identifying the heterogeneity of the TB incidence can lead to informed targeted public health responses, making it an attractive approach [16, 57, 58] for advancing towards the eradication of TB. These effects of a stratified heterogeneity can improve the efficiency of targeting high-risk groups based on the spatial location and socioeconomic determinants [58]. The q value represents the stratified heterogeneity of the TB incidence among the stratified countries by the country type and development stage in the Geotree of TB incidence. First, most of Asia, most of eastern Europe, and all of sub-Saharan Africa had a high TB burden (Fig. 1).

In our study, low-income and lower-middle-income countries were more vulnerable to TB (Figs. 1 and 2). This result is consistent with the results of previous studies demonstrating that the TB incidence displays geographic clustering at multiple resolutions [59]. However, the average TB incidence rate in type I countries is mildly inferior to that in type II countries (Fig. 2) because several high-TB burden countries are lower-middle-income countries [60] (e.g., India, Indonesia, Pakistan, and Tanzania).

The development of TB is relatively low in high development countries. The average TB incidence rates demonstrated a marked declining trend (Fig. 3) from 2010 to 2019, which is in accordance with the above results suggesting a negative association with GDP per capita. Meanwhile, the average rates of change in the TB incidence in type I and type II countries are slightly lower than those in type III and type IV countries, which is because the development assistance growth rate for TB has substantially showed an deceleration since 2010 [61]. This will give rise to more challenges for health systems to reduce the burden of TB in type I and II countries than in type III and IV countries [9, 61, 62].

Second, the average TB incidence exhibited different trends in different country development stages. The average TB incidence decreases as the development stage increased (Fig. 4). As the development stage increases, a country typically experiences improvements in better living conditions, healthcare access, improved education [6]. Countries in high development stage typically have better healthcare access, enabling earlier diagnosis and treatment of TB cases, which can prevent disease transmission. Improved living conditions, including better sanitation, housing, and nutrition, associated with higher development stage also reduce the risk of TB infection and transmission [19, 63]. Additionally, higher education levels in these countries promote awareness and understanding of TB prevention and treatment, further contributing to lower TB incidence rates [63]. Furthermore, as the development stage increases, access to antiretroviral therapy (ART) for HIV-positive individuals also increases, reducing their risk of TB infection as HIV is a significant risk factor for TB [64].However, the average TB incidence increased from stage 1 to stage 2 development stage. This was attributed to the fact that the transition from development stage 1 to development stage 2 only occurred in type I and II countries with a high-burden of TB, where TB is endemic, where health and surveillance systems are weak and underdiagnosis and underreporting cannot be avoided [65].

At the same time, the average TB incidence increased within development stages the fastest from 2010 to 2019 in development stage 5 (Fig. 5). This was due to the migration from countries with high TB incidence to the those with a low or medium TB incidence (< 40 per 100,000 population) (stage 5) [66, 67].

Finally, combining the TB incidence map with the evolutionary Geotree sheds light on the phenomenon of “hot spots” and spatial stratified heterogeneity of countries, as shown in Fig. 1. Hot spots were observed at the bottom of the tree. For example, a high TB incidence rate was observed in South Africa, the Philippines, Lesotho, Gabon, and Central African Republic, which were highest at over 500 (per 100,000 population) in 2010 and 2019 at the bottom of Geotree. There is a high level of stratified heterogeneity between the top and bottom of the “tree” structure (Fig. 1).

Despite the importance and necessity of the directly observed treatment, short course strategy in reducing the TB incidence, socioeconomic factors play a significant role in the control of TB. Understanding a country’s TB status in the context of its socioeconomic position represents crucial input to a TB control policy. There is a clear correlation between socioeconomic factors and TB burden, and there is significant stratified heterogeneity in the distribution of TB incidence according to socioeconomic structure (country type and development stage).

The socioeconomic determinants (GDP per capita, urbanization rate, and SDI) have inhibiting effects on TB incidence (Table 1), which agrees with previously reported results [55, 56, 68, 69]. This is mainly because these factors reflect socioeconomic patterns that are linked to economic development, health services, education and income level in ways that have important implications for TB infection control. GeoDetector was used to detect the driving socioeconomic factors of TB incidence. The impact of the stratified heterogeneity of the TB incidence is mainly influenced by socioeconomic determinants, especially by the SDI in our study, which interpreted the highest stratified heterogeneity of the TB incidence. The SDI is calculated as the geometric mean of income per capita, total fertility, and average educational attainment [70, 71].

In this study, the evolutionary pathway of the TB incidence can be described as the epitaxial growth of a region through an expression of the Markov chain (Fig. 6), which is used to quantify the regional rate of change in the TB incidence. The Markov chain analysis can present the evolutionary way of a region that has transitioned into a higher development stage, compensating for time. The highest average rate of change in the TB incidence was up to -45.24% in stage 4 for type IV countries, and 37 countries showed an average change rate of -13.93% in stage 5 for type IV countries, which explained socioeconomic progress dampened the increase in the TB incidence [72]. In contrast, the lowest rate of change in the TB incidence was -4.78% in stage 3 of type II countries, which may be caused by poor socioeconomic development, poor health services, and decreased development assistance [61, 65, 72]. The Markov chain analysis represents the change in the country development stage, which is consistent with this phenomenon that, as the tree grows, the leaves advance to the next position in Geotree, as shown in Fig. 1.

The TB incidence trend in 2030 was predicted by embedding the MLM model into Geotree model on the basis of the hierarchical results of the TB incidence Geotree constructed for 2019 (Fig. 7 and Supplementary file 2. Table S1). According to the forecast, the overall incidence rate is expected to significantly decrease in 2030. Lower development stage countries would experience the highest incidence rates. Specifically, the incidence rates are highest in low-middle development stage countries. This may be due to the fact that several high-TB burden countries are low-middle development stage countries (e.g., India, Pakistan, Bangladesh, and Tanzania) [73]. By this estimation, 26 of 173 countries would eradicate TB. Of these, 20 out of 26 countries are in high development stage countries. Progress needs to be accelerated to fulfil the “End” “TB” target, which includes improving the diagnosis and treatment, raising awareness about TB prevention, scaling up interventions to mitigate risk factors, and integrating HIV control programs, especially in lower development stage countries. The performance of the MLM is better than that of the GLR model, which indicates that hierarchical heterogeneity exists in the distribution of TB. At the same time, learning which countries lag behind the trajectory of socioeconomic development for those measures can guide investments and intervention efforts to meet the SDG target by 2030 [4, 62, 65].

According to our existing research, our findings do have some guiding significance. To eliminate TB, countries of the same at similar development stage or within the same type can draw on the experiences of countries at higher development stages that are tailored to their unique characteristics. Of course, the recommendation to learn from higher development stage countries is not intended to be prescriptive or one-size-fits-all, but rather a starting point for further exploration and adaptation to local contexts.

This study tentatively adopted the Geotree model in combination with a Markov chain analysis and the MLM to conceptualize and examine the complex geospatial temporal processes of TB, including past and future mechanisms, which is of great significance to TB prevention throughout the world.

Nevertheless, there are some limitations to this study. Because of the limitation of data, only 173 countries territories were considered to explore the geographical evolutionary pathway of global tuberculosis incidence trends. In the future, more data would be collected. Furthermore, the absence of global socio-economic indicators utilizing countries as statistical units necessitated the use of only publicly available data indicators for the purpose of Geotree classification. This approach somewhat diminishes the advantages of the multidimensional coordinate system employed by Geotree. In the future, the socioeconomic indicators would be collected as much as possible to improve the construction of TB incidence Geotree.

## Conclusion

TB incidence decrease varies in the world [74] in the context of health and human development. TB incidence presents obvious stratified heterogeneity in different country types and development stages, which can help achieve a maximum level of TB care and control. That is visualized and modeled by the Geotree method. The key to success is related to the capacity to learn the geographical evolutionary pathway of TB. TB incidence and determinants would evolve along a branch of a type from the lower to higher countries; countries on the same type or development stage can learn the TB control experience from countries with higher types or development stages to formulate effective policies and plans based on their own characteristics. We predict that the TB incidence will be 91.581 (per 100,000 population) along the current path.

## Supplementary Information

**Additional file 1: Fig. S1.** Socioeconomic drivers influencing the prevalence of TB and their proxy variables. **Fig. S2.** Geotree of TB incidence. Note: The first branches, the second trunks and the leaves represent country types, country development stages and countries, respectively. **Table S1.** Countries (173 countries and regions) belong to type I to IV. **Table S2.** Indicators of country development stages in 173 countries and territories in 2010 and 2019. **Table S3.** Countries belong to development stage 1 to 5 in 2010. **Table S4.** Countries belong to development stage 1 to 5 in 2019.

**Additional file 2: Table S1.** Incidence of tuberculosis (per 10,0000 population) in 173 countries and territories in 2030. Note: A negative value represents the elimination of tuberculosis.

## Data Availability

The TB data the study used was from WHO database (https://www.who.int/data/gho/data/indicators/indicator-details/GHO/incidence-of-tuberculosis-(per-100-000-population-per-year)) and the data was publicly accessible. The other data were from the World Bank database, GBD, and SSP database, and they were publicly accessible.

## Abbreviations

TB: Tuberculosis
GDP: Gross domestic product
SDI: Socio-demographic index
LMICs: Low- and middle-income countries
MLM: Multilevel model
GBD: Global Burden of Disease Study
GLR: Global linear regression
SDGs: Sustainable development goals
WHO: World Health Organization

## References

[1] Vos T, Lim SS, Abbafati C, Abbas KM, Abbasi M, Abbasifard M, Abbasi-Kangevari M, Abbastabar H, Abd-Allah F, Abdelalim A (2020). Global burden of 369 diseases and injuries in 204 countries and territories, 1990–2019: a systematic analysis for the Global Burden of Disease Study 2019. The Lancet.

[2] Floyd K, Glaziou P, Zumla A, Raviglione M (2018). The global tuberculosis epidemic and progress in care, prevention, and research: an overview in year 3 of the End TB era. Lancet Respir Med.

[3] Castro KG, Colvin CE (2018). Updated global tuberculosis targets: a welcome ambition in need of attention to quality of care. Int J Tuberc Lung Dis.

[4] Lönnroth K, Raviglione M (2016). The WHO's new End TB Strategy in the post-2015 era of the Sustainable Development Goals. Trans R Soc Trop Med Hyg.

[5] Murray CJ, Ortblad KF, Guinovart C, Lim SS, Wolock TM, Roberts DA, Dansereau EA, Graetz N, Barber RM, Brown JC (2014). Global, regional, and national incidence and mortality for HIV, tuberculosis, and malaria during 1990–2013: a systematic analysis for the Global Burden of Disease Study 2013. Lancet.

[6] Abubakar I, Tillmann T, Banerjee A. Global, regional, and national age-sex specific all-cause and cause-specific mortality for 240 causes of death, 1990–2013: a systematic analysis for the Global Burden of Disease Study 2013. Lancet. 2015;385(9963):117–71. 10.1016/S0140-6736(14)61682-2 PMC4340604 25530442

[7] Wang H, Naghavi M, Allen C, Barber RM, Bhutta ZA, Carter A, Casey DC, Charlson FJ, Chen AZ, Coates MM (2016). Global, regional, and national life expectancy, all-cause mortality, and cause-specific mortality for 249 causes of death, 1980–2015: a systematic analysis for the Global Burden of Disease Study 2015. The Lancet.

[8] Floyd K, Glaziou P, Houben R, Sumner T, White RG, Raviglione M (2018). Global tuberculosis targets and milestones set for 2016–2035: definition and rationale. Int J Tuberc Lung Dis.

[9] Dye C, Lönnroth K, Jaramillo E, Williams BG, Raviglione M (2009). Trends in tuberculosis incidence and their determinants in 134 countries. Bull World Health Organ.

[10] Kyu HH, Maddison ER, Henry NJ, Ledesma JR, Wiens KE, Reiner R, Biehl MH, Shields C, Osgood-Zimmerman A, Ross JM (2018). Global, regional, and national burden of tuberculosis, 1990–2016: results from the Global Burden of Diseases, Injuries, and Risk Factors 2016 Study. Lancet Infect Dis.

[11] MacNeil A, Glaziou P, Sismanidis C, Date A, Maloney S, Floyd K (2020). Global epidemiology of tuberculosis and progress toward meeting global targets—worldwide, 2018. Morb Mortal Wkly Rep.

[12] Cao K, Yang K, Wang C, Guo J, Tao L, Liu Q, Gehendra M, Zhang Y, Guo X (2016). Spatial-temporal epidemiology of tuberculosis in mainland China: an analysis based on Bayesian theory. Int J Environ Res Public Health.

[13] Jafari-Koshki T, Arsang-Jang S, Raei M (2015). Applying spatiotemporal models to study risk of smear-positive tuberculosis in Iran, 2001–2012. Int J Tuberc Lung Dis.

[14] Alene KA, Viney K, McBryde ES, Clements AC (2017). Spatial patterns of multidrug resistant tuberculosis and relationships to socio-economic, demographic and household factors in northwest Ethiopia. PLoS ONE.

[15] Beiranvand R, Karimi A, Delpisheh A, Sayehmiri K, Soleimani S, Ghalavandi S (2016). Correlation assessment of climate and geographic distribution of tuberculosis using geographical information system (GIS). Iran J Public Health.

[16] Trauer JM, Dodd PJ, Gomes MGM, Gomez GB, Houben RM, McBryde ES, Melsew YA, Menzies NA, Arinaminpathy N, Shrestha S (2019). The importance of heterogeneity to the epidemiology of tuberculosis. Clin Infect Dis.

[17] Wang JF, Liu XH, Peng L, Chen HY, Driskell L, Zheng XY (2012). Cities evolution tree and applications to predicting urban growth. Popul Environ.

[18] Zhou Y, Poon J, Yang Y (2021). China's CO2 emission intensity and its drivers: an evolutionary Geo-Tree approach. Resour Conserv Recycl.

[19] Lienhardt C, Glaziou P, Uplekar M, Lönnroth K, Getahun H, Raviglione M (2012). Global tuberculosis control: lessons learnt and future prospects. Nat Rev Microbiol.

[20] Wang L, Xu C, Hu M, Qiao J, Chen W, Li T, Qian S, Yan M (2021). Spatio-temporal variation in tuberculosis incidence and risk factors for the disease in a region of unbalanced socio-economic development. BMC Public Health.

[21] Pedrazzoli D, Boccia D, Dodd PJ, Lönnroth K, Dowdy D, Siroka A, Kimerling M, White R, Houben R (2017). Modelling the social and structural determinants of tuberculosis: opportunities and challenges. Int J Tuberc Lung Dis.

[22] Martins N, Kelly PM, Grace JA, Zwi AB (2006). Reconstructing tuberculosis services after major conflict: experiences and lessons learned in East Timor. PLoS Med.

[23] Cuevas LE, Yassin MA, Al-Sonboli N, Lawson L, Arbide I, Al-Aghbari N, Bahadur Sherchand J, Al-Absi A, Emenyonu EN, Merid Y (2011). A multi-country non-inferiority cluster randomized trial of frontloaded smear microscopy for the diagnosis of pulmonary tuberculosis. PLoS Med.

[24] Lönnroth K, Castro KG, Chakaya JM, Chauhan LS, Floyd K, Glaziou P, Raviglione MC (2010). Tuberculosis control and elimination 2010–50: cure, care, and social development. Lancet.

[25] Hargreaves JR, Boccia D, Evans CA, Adato M, Petticrew M, Porter JDH (2011). The social determinants of tuberculosis: from evidence to action. Am J Public Health.

[26] Rubel AJ, Garro LC (1992). Social and cultural factors in the successful control of tuberculosis. Public Health Rep.

[27] Munch Z, Van Lill S, Booysen C, Zietsman H, Enarson D, Beyers N (2003). Tuberculosis transmission patterns in a high-incidence area: a spatial analysis. Int J Tuberc Lung Dis.

[28] Lin H-H, Ezzati M, Murray M (2007). Tobacco smoke, indoor air pollution and tuberculosis: a systematic review and meta-analysis. PLoS Med.

[29] Sonnenberg P, Glynn JR, Fielding K, Murray J, Godfrey-Faussett P, Shearer S (2005). How soon after infection with HIV does the risk of tuberculosis start to increase? A retrospective cohort study in South African gold miners. J Infect Dis.

[30] Baghaei P, Marjani M, Javanmard P, Tabarsi P, Masjedi MR (2013). Diabetes mellitus and tuberculosis facts and controversies. J Diabetes Metab Disord.

[31] Sun W, Gong J, Zhou J, Zhao Y, Tan J, Ibrahim AN, Zhou Y (2015). A spatial, social and environmental study of tuberculosis in China using statistical and GIS technology. Int J Environ Res Public Health.

[32] Wingfield T, Tovar MA, Huff D, Boccia D, Saunders MJ, Datta S, Montoya R, Ramos E, Lewis JJ, Gilman RH (2016). Beyond pills and tests: addressing the social determinants of tuberculosis. Clin Med.

[33] Randremanana RV, Sabatier P, Rakotomanana F, Randriamanantena A, Richard V (2009). Spatial clustering of pulmonary tuberculosis and impact of the care factors in Antananarivo City. Tropical Med Int Health.

[34] Mutembo S, Mutanga JN, Musokotwane K, Kanene C, Dobbin K, Yao X, Li C, Marconi VC, Whalen CC (2019). Urban-rural disparities in treatment outcomes among recurrent TB cases in Southern Province. Zambia BMC infectious diseases.

[35] de DíazQuijano E, Brugal MT, Pasarín MI, Galdós-Tangüís H, Cayla J, Borrell C (2001). Influence of social inequality, social unrest and extreme poverty on tuberculosis morbidity in the City of Barcelona. Revista Española de Salud Pública.

[36] World Health Organization:Global tuberculosis report. World Health Organization. [https://www.who.int/teams/global-tuberculosis-programme/data#csv_files]

[37] Hunter JM, Thomas MO (1984). Hypothesis of leprosy, tuberculosis and urbanization in Africa. Soc Sci Med (1982).

[38] The World Bank:GDP per capita (current US$). The World Bank Group. [https://data.worldbank.org/indicator/NY.GDP.PCAP.CD?view=chart]

[39] Kassebaum NJ, Arora M, Barber RM, Bhutta ZA, Brown J, Carter A, Casey DC, Charlson FJ, Coates MM, Coggeshall M. Global, regional, and national disability-adjusted life-years (DALYs) for 315 diseases and injuries and healthy life expectancy (HALE), 1990–2015: a systematic analysis for the Global Burden of Disease Study 2015. Lancet. 2016;388(10053):1603–58. 10.1016/S0140-6736(16)31460-X PMC5388857 27733283

[40] Naghavi M, Abajobir AA, Abbafati C, Abbas KM, Abd-Allah F, Abera SF, Aboyans V, Adetokunboh O, Afshin A, Agrawal A. Global, regional, and national age-sex specific mortality for 264 causes of death, 1980–2016: a systematic analysis for the Global Burden of Disease Study 2016. Lancet. 2017;390(10100):1151–210. 10.1016/S0140-6736(17)32152-9 PMC5605883 28919116

[41] Hay SI, Abajobir AA, Abate KH, Abbafati C, Abbas KM, Abd-Allah F, Abdulkader RS, Abdulle AM, Abebo TA, Abera SF. Global, regional, and national disability-adjusted life-years (DALYs) for 333 diseases and injuries and healthy life expectancy (HALE) for 195 countries and territories, 1990–2016: a systematic analysis for the Global Burden of Disease Study 2016. Lancet. 2017;390(10100):1260–344. 10.1016/S0140-6736(17)32130-X PMC5605707 28919118

[42] Vos T, Abajobir AA, Abate KH, Abbafati C, Abbas KM, Abd-Allah F, Abdulkader RS, Abdulle AM, Abebo TA, Abera SF. Global, regional, and national incidence, prevalence, and years lived with disability for 328 diseases and injuries for 195 countries, 1990–2016: a systematic analysis for the Global Burden of Disease Study 2016. Lancet. 2017;390(10100):1211–59. 10.1016/S0140-6736(17)32154-2 PMC5605509 28919117

[43] Global Burden of Disease Collaborative Network. Global Burden of Disease Study 2019 (GBD 2019) Socio-Demographic Index (SDI) 1950–2019. Seattle, United States of America: Institute for Health Metrics and Evaluation (IHME). 2020.

[44] Wang Y, Wang J. Modelling and prediction of global non-communicable diseases. BMC Public Health. 2020;20:1-13. 10.1186/s12889-020-08890-4 PMC7268487 32487173

[45] He X, Sheng J (2020). New evaluation system for the modernization level of a province or a city based on an improved entropy method. Environ Monit Assess.

[46] United Nations Population Division.Urban population (% of total population). World Urbanization Prospects: 2018 Revision. [https://data.worldbank.org/indicator/SP.URB.TOTL.IN.ZS?view=chart]

[47] Riahi K, Van Vuuren DP, Kriegler E, Edmonds J, O’neill BC, Fujimori S, Bauer N, Calvin K, Dellink R, Fricko O. The Shared Socioeconomic Pathways and their energy, land use, and greenhouse gas emissions implications: an overview. Global Environ Change. 2017;42:153-168.

[48] Nakajima H, Kume A, Ishida M, Ohmiya T, Mizoue N (2011). Evaluation of estimates of crown condition in forest monitoring: comparison between visual estimation and automated crown image analysis. Ann For Sci.

[49] Wang J-F, Zhang T-L, Fu B-J (2016). A measure of spatial stratified heterogeneity. Ecol Ind.

[50] Wang JF, Li XH, Christakos G, Liao YL, Zhang T, Gu X, Zheng XY (2010). Geographical detectors-based health risk assessment and its application in the neural tube defects study of the Heshun Region, China. Int J Geogr Inf Sci.

[51] Goldstein H. Multilevel statistical models, vol. 922. John Wiley & Sons. 2011.

[52] Rasbash J, Goldstein H. Efficient analysis of mixed hierarchical and cross-classified random structures using a multilevel model. J Educ Behav Stat. 1994;19(4):337-50.

[53] Leyland AH, Groenewegen PP (2003). Multilevel modelling and public health policy. Scandinavian J Public Health.

[54] Wang J, Xu C (2017). Geodetector: principle and prospective. Acta Geogr Sin.

[55] Boccia D, Pedrazzoli D, Wingfield T, Jaramillo E, Lönnroth K, Lewis J, Hargreaves J, Evans CA (2016). Towards cash transfer interventions for tuberculosis prevention, care and control: key operational challenges and research priorities. BMC Infect Dis.

[56] Fojo AT, Stennis NL, Azman AS, Kendall EA, Shrestha S, Ahuja SD, Dowdy DW (2017). Current and future trends in tuberculosis incidence in New York City: a dynamic modelling analysis. ancet Public Health.

[57] Cohen T, Colijn C, Finklea B, Murray M (2007). Exogenous re-infection and the dynamics of tuberculosis epidemics: local effects in a network model of transmission. J R Soc Interface.

[58] Dowdy DW, Golub JE, Chaisson RE, Saraceni V (2012). Heterogeneity in tuberculosis transmission and the role of geographic hotspots in propagating epidemics. Proc Natl Acad Sci.

[59] Shaweno D, Karmakar M, Alene KA, Ragonnet R, Clements AC, Trauer JM, Denholm JT, McBryde ES (2018). Methods used in the spatial analysis of tuberculosis epidemiology: a systematic review. BMC Med.

[60] Menzies NA, Bellerose M, Testa C, Swartwood NA, Malyuta Y, Cohen T, Marks SM, Hill AN, Date AA, Maloney SA (2020). Impact of effective global tuberculosis control on health and economic outcomes in the United States. Am J Respir Crit Care Med.

[61] Dieleman J, Campbell M, Chapin A, Eldrenkamp E, Fan VY, Haakenstad A, Kates J, Liu Y, Matyasz T, Micah A (2017). Evolution and patterns of global health financing 1995–2014: development assistance for health, and government, prepaid private, and out-of-pocket health spending in 184 countries. The Lancet.

[62] Kyu HH, Maddison ER, Henry NJ, Mumford JE, Barber R, Shields C, Brown JC, Nguyen G, Carter A, Wolock TM (2018). The global burden of tuberculosis: results from the Global Burden of Disease Study 2015. Lancet Infect Dis.

[63] Lönnroth K, Jaramillo E, Williams BG, Dye C, Raviglione M (2009). Drivers of tuberculosis epidemics: the role of risk factors and social determinants. Soc Sci Med.

[64] Millet J-P, Moreno A, Fina L, Del Baño L, Orcau A, De Olalla PG, Cayla JA (2013). Factors that influence current tuberculosis epidemiology. Eur Spine J.

[65] World Health Organization. Global tuberculosis report 2014. World Health Organization; 2014.

[66] Jackson S, Kabir Z, Comiskey C (2021). Effects of migration on tuberculosis epidemiological indicators in low and medium tuberculosis incidence countries: a systematic review. J Clin Tuberc Other Mycobact Dis.

[67] Abarca Tomás B, Pell C, Bueno Cavanillas A, Guillén Solvas J, Pool R, Roura M (2013). Tuberculosis in migrant populations. A systematic review of the qualitative literature. PLOS one.

[68] Xie M, Liu X, Cao X, Guo M, Li X (2020). Trends in prevalence and incidence of chronic respiratory diseases from 1990 to 2017. Respir Res.

[69] Alcohol GBD, Drug Use C (2018). The global burden of disease attributable to alcohol and drug use in 195 countries and territories, 1990–2016: a systematic analysis for the Global Burden of Disease Study 2016. Lancet Psychiatry.

[70] Collaborators GBDB (2018). Burden of disease in Brazil, 1990–2016: a systematic subnational analysis for the Global Burden of Disease Study 2016. Lancet (London, England).

[71] Naghavi M, Abajobir AA, Abbafati C, Abbas KM, Abd-Allah F, Abera SF, Aboyans V, Adetokunboh O, Afshin A, Agrawal A (2017). Global, regional, and national age-sex specific mortality for 264 causes of death, 1980–2016: a systematic analysis for the Global Burden of Disease Study 2016. Lancet.

[72] Rasanathan K, Sivasankara Kurup A, Jaramillo E, Lönnroth K (2011). The social determinants of health: key to global tuberculosis control. Int J Tuberc Lung Dis.

[73] World Health Organization. On the road to ending TB: highlights from the 30 highest TB burden countries. World Health Organization; 2016.

[74] Lauzardo M, Peloquin CA (2012). Antituberculosis therapy for 2012 and beyond. Expert Opin Pharmacother.

