# Supplementary material for: Geographical evolutionary pathway of global tuberculosis incidence trends
Source: BMC Public Health. 2023 Apr 24;23:755. doi: 10.1186/s12889-023-15553-7 (PMC10123998; doi:10.1186/s12889-023-15553-7)
Supplement: Supplementary file 1 — Additional file 1: Fig. S1. Socioeconomic drivers influencing the prevalence of TB and their proxy variables. Fig. S2. Geotree of TB incidence. Note: The first branches, the second trunks and the leaves represent country types, country development stages and countries, respectively. Table S1. Countries (173 countries and regions) belong to type I to IV. Table S2. Indicators of country development stages in 173 countries and territories in 2010 and 2019. Table S3. Countries belong to development stage 1 to 5 in 2010. Table S4. Countries belong to development stage 1 to 5 in 2019. [file 12889_2023_15553_MOESM1_ESM.docx]

**
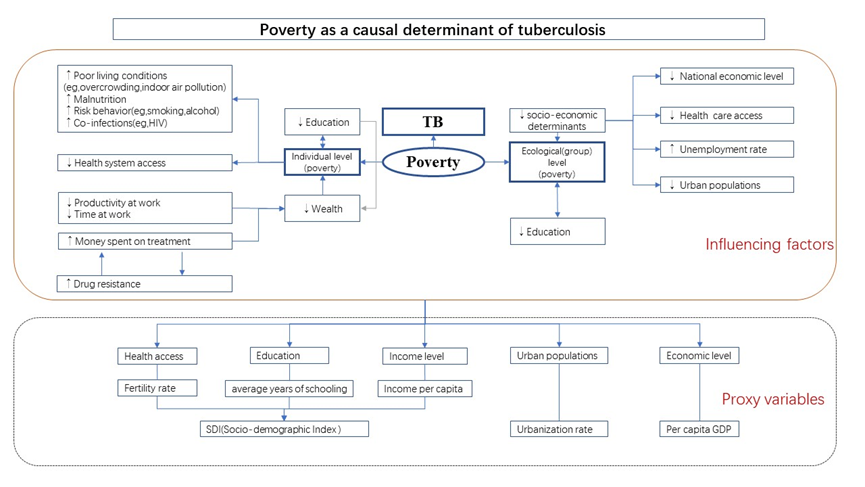
**

**Fig. S1 Socioeconomic drivers influencing the prevalence of TB and their proxy variables**


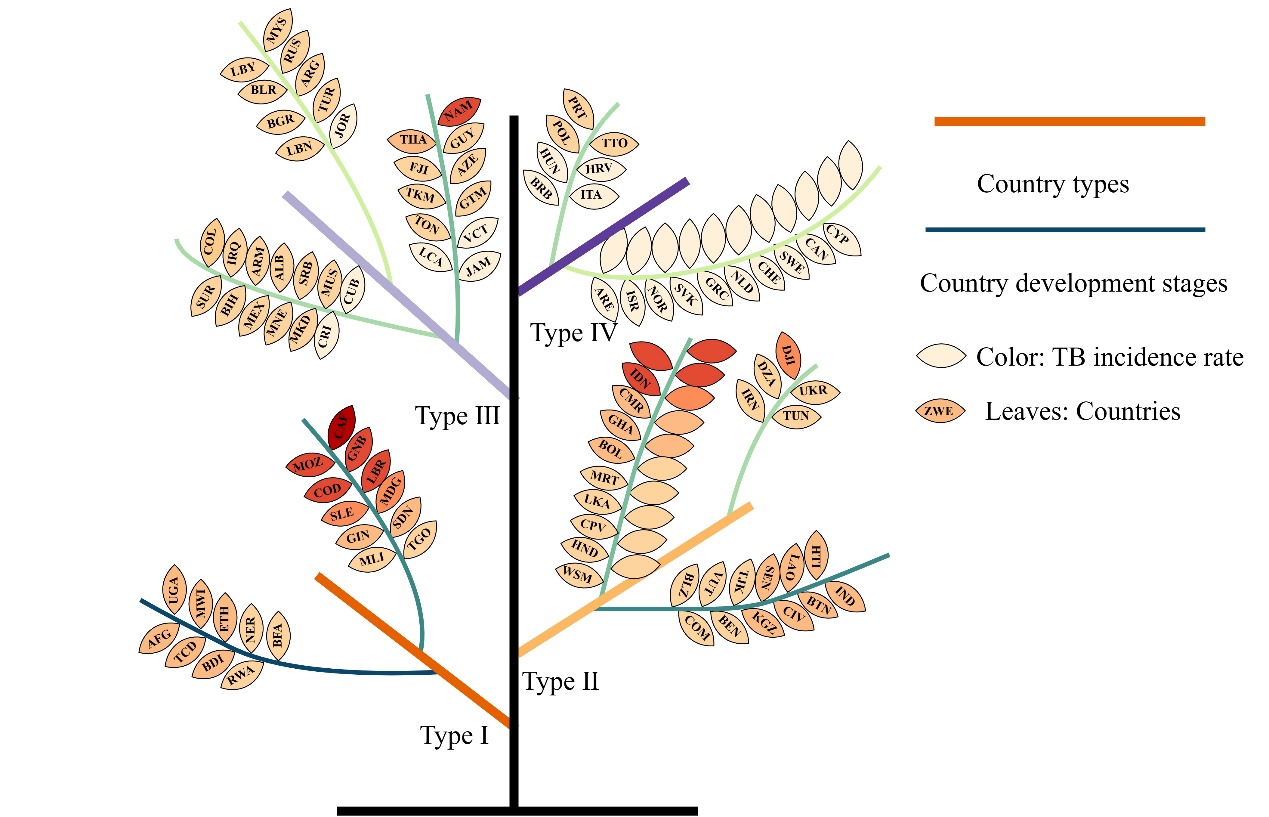
**Fig. S2** Geotree of TB incidence. Note: The first branches, the second trunks and the leaves represent country types, country development stages and countries, respectively.

**Table S1** Countries (173 countries and regions) belong to type I to IV

| **Country type** | **Country name** |
| --- | --- |
| **Type I** | Afghanistan, Burkina Faso, Burundi, Central African Republic, Chad, Congo, Dem. Rep., Ethiopia, Guinea, Guinea-Bissau, Liberia, Madagascar, Malawi, Mali, Mozambique, Niger, Rwanda, Sierra Leone, Sudan, Syrian Arab Republic, The Gambia, Togo, Uganda |
| **Type II** | Algeria, Angola, Bangladesh, Belize, Benin, Bhutan, Bolivia, Cambodia, Cameroon, Cape Verde, Comoros, Congo, Rep., Cote d'Ivoire, Djibouti, Egypt, Arab Rep., El Salvador, Eswatini, Ghana, Haiti, Honduras, India, Indonesia, Iran, Islamic Rep., Kenya, Kyrgyz Republic, Lao PDR, Lesotho, Mauritania, Mongolia, Morocco, Myanmar, Nepal, Nicaragua, Nigeria, Pakistan, Papua New Guinea, Philippines, Samoa, Sao Tome and Principe, Senegal, Solomon Islands, Sri Lanka, Tajikistan, Tanzania, Timor-Leste, Tunisia, Ukraine, Uzbekistan, Vanuatu, Vietnam, Zambia, Zimbabwe |
| **Type III** | Albania, Argentina, Armenia, Azerbaijan, Belarus, Bosnia and Herzegovina, Botswana, Brazil, Bulgaria, China, Colombia, Costa Rica, Cuba, Dominican Republic, Ecuador, Equatorial Guinea, Fiji, Gabon, Georgia, Guatemala, Guyana, Iraq, Jamaica, Jordan, Kazakhstan, Lebanon, Libya, Malaysia, Maldives, Mauritius, Mexico, Moldova, Montenegro, Namibia, North Macedonia, Panama, Paraguay, Peru, Romania, Russian Federation, St. Lucia, St. Vincent and the Grenadines, Serbia, South Africa, Suriname, Thailand, Tonga, Turkey, Turkmenistan |
| **Type IV** | Australia, Austria, Bahrain, Barbados, Belgium, Brunei Darussalam, Canada, Chile, Croatia, Cyprus, Czech Republic, Denmark, Estonia, Finland, France, Germany, Greece, Hungary, Iceland, Ireland, Israel, Italy, Japan, Kuwait, Latvia, Lithuania, Luxembourg, Malta, Netherlands, New Zealand, Norway, Oman, Poland, Portugal, Puerto Rico, Qatar, Saudi Arabia, Singapore, Slovak Republic, Slovenia, South Korea, Spain, Sweden, Switzerland, The Bahamas, Trinidad and Tobago, United Kingdom, United Arab Emirates, Uruguay, United States |


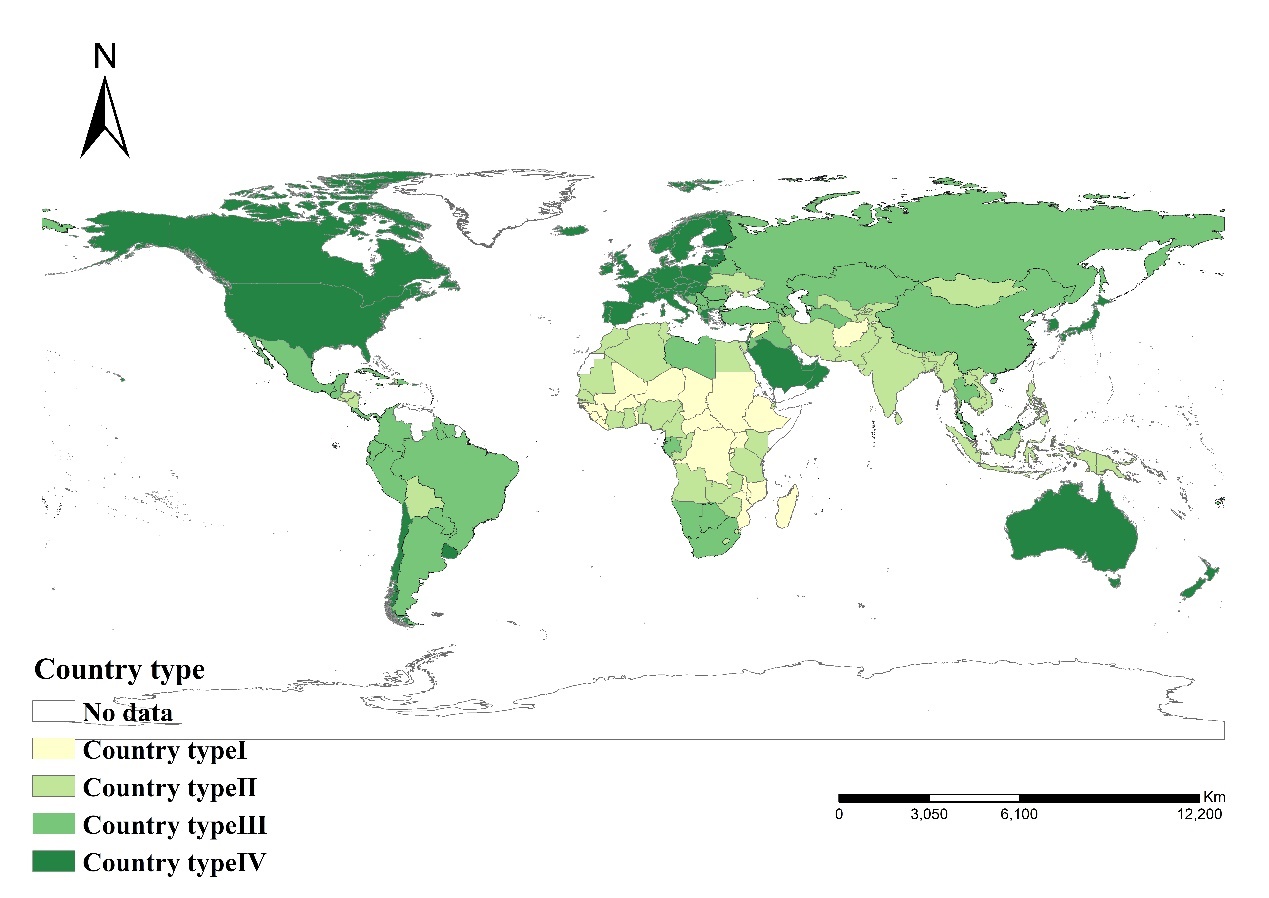


**Fig S3.** Spatial distribution of the four country types

**Table S2** Indicators of country development stages in 173 countries and territories in 2010 and 2019

| **Country development stages** | **SDI** (income per capita, average years of schooling, and total fertility rate) | **Urban population** (% of the total population) |
| --- | --- | --- |
| **1** | <0.455 | <30 |
| **2** | 0.455~0.608 | 30~50 |
| **3** | 0.608~0.690 | 50~60 |
| **4** | 0.690~0.805 | 60~75 |
| **5** | ≥0.805 | ≥75 |

**Table S3** Countries belong to development stage 1 to 5 in 2010

| **Country development stage** | **Country name** |
| --- | --- |
| **Stage 1** | Afghanistan, Burkina Faso, Burundi, Cambodia, Chad, Comoros, Ethiopia, Kenya, Lesotho, Malawi, Myanmar, Nepal, Niger, Papua New Guinea, Rwanda, Solomon Islands, Tanzania, Uganda, Vanuatu |
| **Stage 2** | Angola, Bangladesh, Belize, Benin, Bhutan, Cameroon, Central African Republic, Cote d'Ivoire, Congo, Dem. Rep., Egypt, Arab Rep., Eswatini, Guatemala, Guinea, Guinea-Bissau, Guyana, Haiti, Honduras, India, Indonesia, Kyrgyz Republic, Lao PDR, Liberia, Madagascar, Maldives, Mali, Mauritania, Mozambique, Namibia, Nigeria, Pakistan, Philippines, St. Vincent and the Grenadines, Senegal, Sierra Leone, Sudan, Tajikistan, The Gambia, Timor-Leste, Togo, Tonga, Vietnam, Zambia, Zimbabwe |
| **Stage 3** | Albania, Algeria, Azerbaijan, Bolivia, Bosnia and Herzegovina, Botswana, Cape Verde, China, Congo, Rep., Djibouti, Dominican Republic, Ecuador, El Salvador, Equatorial Guinea, Fiji, Georgia, Ghana, Iraq, Jamaica, Kazakhstan, Mauritius, Moldova, Mongolia, Morocco, Nicaragua, Paraguay, St. Lucia, Samoa, Sao Tome and Principe, Sri Lanka, Suriname, Syrian Arab Republic, Thailand, Turkmenistan, Uzbekistan |
| **Stage 4** | Argentina, Armenia, Barbados, Belarus, Brazil, Brunei Darussalam, Bulgaria, Colombia, Costa Rica, Croatia, Cuba, Estonia, Gabon, Hungary, Iran, Islamic Rep., Italy, Jordan, Latvia, Lebanon, Lithuania, Malaysia, Mexico, Montenegro, North Macedonia, Panama, Peru, Poland, Portugal, Romania, Russian Federation, Serbia, Slovak Republic, South Africa, Trinidad and Tobago, Tunisia, Turkey, Ukraine, Uruguay |
| **Stage 5** | Australia, Austria, Bahrain, Belgium, Canada, Chile, Cyprus, Czech Republic, Denmark, Finland, France, Germany, Greece, Iceland, Ireland, Israel, Japan, Kuwait, Libya, Luxembourg, Malta, Netherlands, New Zealand, Norway, Oman, Puerto Rico, Qatar, Saudi Arabia, Singapore, Slovenia, South Korea, Spain, Sweden, Switzerland, The Bahamas, United Kingdom, United Arab Emirates, United States |

**Table S4** Countries belong to development stage 1 to 5 in 2019

| **Country type** | **Country name** |
| --- | --- |
| **Stage 1** | Afghanistan, Burkina Faso, Burundi, Chad, Ethiopia, Malawi, Nepal, Niger, Papua New Guinea, Rwanda, Solomon Islands, Uganda |
| **Stage 2** | Bangladesh, Belize, Benin, Bhutan, Cambodia, Central African Republic, Comoros, Cote d'Ivoire, Congo, Dem. Rep., Eswatini, Guinea, Guinea-Bissau, Haiti, India, Kenya, Kyrgyz Republic, Lao PDR, Lesotho, Liberia, Madagascar, Maldives, Mali, Mozambique, Myanmar, Pakistan, Senegal, Sierra Leone, Sudan, Tajikistan, Tanzania, Timor-Leste, Togo, Vanuatu, Zambia, Zimbabwe |
| **Stage 3** | Angola, Azerbaijan, Bolivia, Cameroon, Cape Verde, Congo, Rep., Egypt, Arab Rep., El Salvador, Fiji, Ghana, Guatemala, Guyana, Honduras, Indonesia, Jamaica, Mauritania, Mongolia, Morocco, Namibia, Nicaragua, Nigeria, Philippines, St. Lucia, St. Vincent and the Grenadines, Samoa, Sao Tome and Principe, Sri Lanka, Syrian Arab Republic, Thailand, The Gambia, Tonga, Turkmenistan, Uzbekistan, Vietnam |
| **Stage 4** | Albania, Algeria, Armenia, Barbados, Bosnia and Herzegovina, Botswana, Brazil, China, Colombia, Costa Rica, Croatia, Cuba, Djibouti, Dominican Republic, Ecuador, Equatorial Guinea, Gabon, Georgia, Hungary, Iran, Islamic Rep., Iraq, Italy, Kazakhstan, Mauritius, Mexico, Moldova, Montenegro, North Macedonia, Panama, Paraguay, Peru, Poland, Portugal, Romania, Serbia, South Africa, Suriname, Trinidad and Tobago, Tunisia, Ukraine |
| **Stage 5** | Argentina, Australia, Austria, Bahrain, Belarus, Belgium, Brunei Darussalam, Bulgaria, Canada, Chile, Cyprus, Czech Republic, Denmark, Estonia, Finland, France, Germany, Greece, Iceland, Ireland, Israel, Japan, Jordan, Kuwait, Latvia, Lebanon, Libya, Lithuania, Luxembourg, Malaysia, Malta, Netherlands, New Zealand, Norway, Oman, Puerto Rico, Qatar, Russian Federation, Saudi Arabia, Singapore, Slovak Republic, Slovenia, South Korea, Spain, Sweden, Switzerland, The Bahamas, Turkey, United Kingdom, United Arab Emirates, Uruguay, United States |
