# Supplementary material for: Geographical evolutionary pathway of global tuberculosis incidence trends
Source: BMC Public Health. 2023 Apr 24;23:755. doi: 10.1186/s12889-023-15553-7 (PMC10123998; doi:10.1186/s12889-023-15553-7)
Supplement: Supplementary file 2 — Additional file 2: Table S1. Incidence of tuberculosis (per 10,0000 population) in 173 countries and territories in 2030. Note: A negative value represents the elimination of tuberculosis. [file 12889_2023_15553_MOESM2_ESM.docx]

**Table S1** Incidence of tuberculosis (per 10,0000 population) in 173 countries and territories in 2030. Note: A negative value represents the elimination of tuberculosis.

| **ID** | **Country_code** | **Incidence (per 10,0000 population)** |
| --- | --- | --- |
| **1** | **AFG** | **107.26** |
| **2** | **ALB** | **55.09** |
| **3** | **DZA** | **106.71** |
| **4** | **AGO** | **43.25** |
| **5** | **ARG** | **82.05** |
| **6** | **ARM** | **53.97** |
| **7** | **AUS** | **6.99** |
| **8** | **AUT** | **5.78** |
| **9** | **AZE** | **31.78** |
| **10** | **BHR** | **28.47** |
| **11** | **BGD** | **173.67** |
| **12** | **BRB** | **40.28** |
| **13** | **BLR** | **23.93** |
| **14** | **BEL** | **3.43** |
| **15** | **BLZ** | **160.15** |
| **16** | **BEN** | **228.07** |
| **17** | **BTN** | **178.83** |
| **18** | **BOL** | **144.75** |
| **19** | **BIH** | **58.68** |
| **20** | **BWA** | **78.65** |
| **21** | **BRA** | **73.83** |
| **22** | **BRN** | **6.32** |
| **23** | **BGR** | **34.28** |
| **24** | **BFA** | **174.93** |
| **25** | **BDI** | **159.67** |
| **26** | **KHM** | **173.39** |
| **27** | **CMR** | **140.09** |
| **28** | **CAN** | **-0.83** |
| **29** | **CPV** | **138.66** |
| **30** | **CAF** | **231.00** |
| **31** | **TCD** | **171.31** |
| **32** | **CHL** | **27.34** |
| **33** | **CHN** | **51.96** |
| **34** | **COL** | **68.81** |
| **35** | **COM** | **194.67** |
| **36** | **COG** | **121.17** |
| **37** | **CRI** | **68.76** |
| **38** | **CIV** | **211.03** |
| **39** | **HRV** | **20.34** |
| **40** | **CUB** | **61.47** |
| **41** | **CYP** | **22.20** |
| **42** | **CZE** | **32.23** |
| **43** | **DNK** | **-2.05** |
| **44** | **DJI** | **142.88** |
| **45** | **DOM** | **56.92** |
| **46** | **COD** | **117.53** |
| **47** | **ECU** | **69.81** |
| **48** | **EGY** | **99.05** |
| **49** | **SLV** | **139.87** |
| **50** | **GNQ** | **18.37** |
| **51** | **EST** | **1.11** |
| **52** | **SWZ** | **157.30** |
| **53** | **ETH** | **108.22** |
| **54** | **FJI** | **85.14** |
| **55** | **FIN** | **0.96** |
| **56** | **FRA** | **10.62** |
| **57** | **GAB** | **67.45** |
| **58** | **GEO** | **38.00** |
| **59** | **DEU** | **-13.16** |
| **60** | **GHA** | **117.47** |
| **61** | **GRC** | **29.68** |
| **62** | **GTM** | **118.93** |
| **63** | **GIN** | **202.29** |
| **64** | **GNB** | **190.78** |
| **65** | **GUY** | **88.70** |
| **66** | **HTI** | **211.43** |
| **67** | **HND** | **159.44** |
| **68** | **HUN** | **42.44** |
| **69** | **ISL** | **1.89** |
| **70** | **IND** | **135.49** |
| **71** | **IDN** | **99.02** |
| **72** | **IRN** | **100.50** |
| **73** | **IRQ** | **31.52** |
| **74** | **IRL** | **-2.74** |
| **75** | **ISR** | **25.40** |
| **76** | **ITA** | **33.09** |
| **77** | **JAM** | **81.00** |
| **78** | **JPN** | **0.19** |
| **79** | **JOR** | **38.11** |
| **80** | **KAZ** | **46.65** |
| **81** | **KEN** | **170.69** |
| **82** | **KWT** | **-13.74** |
| **83** | **KGZ** | **148.00** |
| **84** | **LAO** | **163.89** |
| **85** | **LVA** | **8.95** |
| **86** | **LBN** | **33.98** |
| **87** | **LSO** | **175.07** |
| **88** | **LBR** | **149.33** |
| **89** | **LBY** | **71.98** |
| **90** | **LTU** | **-10.94** |
| **91** | **LUX** | **-13.48** |
| **92** | **MDG** | **174.03** |
| **93** | **MWI** | **112.86** |
| **94** | **MYS** | **47.64** |
| **95** | **MDV** | **140.89** |
| **96** | **MLI** | **220.32** |
| **97** | **MLT** | **21.79** |
| **98** | **MRT** | **142.15** |
| **99** | **MUS** | **50.33** |
| **100** | **MEX** | **81.23** |
| **101** | **MDA** | **31.00** |
| **102** | **MNG** | **128.99** |
| **103** | **MNE** | **12.10** |
| **104** | **MAR** | **127.48** |
| **105** | **MOZ** | **194.90** |
| **106** | **MMR** | **152.63** |
| **107** | **NAM** | **83.30** |
| **108** | **NPL** | **141.87** |
| **109** | **NLD** | **-4.33** |
| **110** | **NZL** | **8.22** |
| **111** | **NIC** | **163.38** |
| **112** | **NER** | **201.16** |
| **113** | **NGA** | **122.59** |
| **114** | **MKD** | **44.15** |
| **115** | **NOR** | **-14.34** |
| **116** | **OMN** | **11.52** |
| **117** | **PAK** | **184.35** |
| **118** | **PAN** | **60.31** |
| **119** | **PNG** | **168.34** |
| **120** | **PRY** | **75.82** |
| **121** | **PER** | **70.11** |
| **122** | **PHL** | **113.74** |
| **123** | **POL** | **28.43** |
| **124** | **PRT** | **45.96** |
| **125** | **PRI** | **9.50** |
| **126** | **QAT** | **-2.66** |
| **127** | **ROU** | **39.29** |
| **128** | **RUS** | **13.15** |
| **129** | **RWA** | **93.88** |
| **130** | **LCA** | **53.47** |
| **131** | **VCT** | **87.58** |
| **132** | **WSM** | **116.59** |
| **133** | **STP** | **130.94** |
| **134** | **SAU** | **-5.97** |
| **135** | **SEN** | **214.72** |
| **136** | **SRB** | **27.19** |
| **137** | **SLE** | **169.55** |
| **138** | **SGP** | **-0.35** |
| **139** | **SVK** | **25.74** |
| **140** | **SVN** | **13.09** |
| **141** | **SLB** | **161.19** |
| **142** | **ZAF** | **64.18** |
| **143** | **KOR** | **2.69** |
| **144** | **ESP** | **35.73** |
| **145** | **LKA** | **85.01** |
| **146** | **SDN** | **116.48** |
| **147** | **SUR** | **77.40** |
| **148** | **SWE** | **4.85** |
| **149** | **CHE** | **-21.91** |
| **150** | **SYR** | **96.62** |
| **151** | **TJK** | **140.05** |
| **152** | **TZA** | **191.41** |
| **153** | **THA** | **70.49** |
| **154** | **BHS** | **21.21** |
| **155** | **GMB** | **144.60** |
| **156** | **TLS** | **153.46** |
| **157** | **TGO** | **173.23** |
| **158** | **TON** | **87.76** |
| **159** | **TTO** | **29.09** |
| **160** | **TUN** | **101.22** |
| **161** | **TUR** | **25.01** |
| **162** | **TKM** | **53.76** |
| **163** | **UGA** | **102.71** |
| **164** | **GBR** | **4.60** |
| **165** | **UKR** | **70.84** |
| **166** | **ARE** | **7.45** |
| **167** | **URY** | **47.52** |
| **168** | **USA** | **-7.73** |
| **169** | **UZB** | **103.47** |
| **170** | **VUT** | **186.08** |
| **171** | **VNM** | **107.93** |
| **172** | **ZMB** | **143.09** |
| **173** | **ZWE** | **211.61** |
